# COVID-19-associated coagulopathy and acute kidney injury in critically ill patients

**DOI:** 10.31744/einstein_journal/2023AO0119

**Published:** 2023-08-30

**Authors:** Bruno Caldin da Silva, Ricardo Luiz Cordioli, Bento Fortunato Cardoso dos Santos, João Carlos de Campos Guerra, Roseny dos Reis Rodrigues, Guilherme Martins de Souza, Carolina Ashihara, Thais Dias Midega, Niklas Söderberg Campos, Bárbara Vieira Carneiro, Flávia Nunes Dias Campos, Hélio Penna Guimarães, Gustavo Faissol Janot de Matos, Valdir Fernandes de Aranda, Leonardo José Rolim Ferraz, Thiago Domingos Corrêa

**Affiliations:** 1 Hospital Israelita Albert Einstein São Paulo SP Brazil Hospital Israelita Albert Einstein, São Paulo, SP, Brazil.

**Keywords:** Acute kidney injury, COVID-19, SARS-CoV-2, Coronavirus infections, Thrombosis, Blood coagulation, Intensive care units

## Abstract

**Objective:**

The incidence of thrombotic events and acute kidney injury is high in critically ill patients with COVID-19. We aimed to evaluate and compare the coagulation profiles of patients with COVID-19 developing acute kidney injury *versus* those who did not, during their intensive care unit stay.

**Methods:**

Conventional coagulation and platelet function tests, fibrinolysis, endogenous inhibitors of coagulation tests, and rotational thromboelastometry were conducted on days 0, 1, 3, 7, and 14 following intensive care unit admission.

**Results:**

Out of 30 patients included, 13 (43.4%) met the criteria for acute kidney injury. Comparing both groups, patients with acute kidney injury were older: 73 (60-84) *versus* 54 (47-64) years, p=0.027, and had a lower baseline glomerular filtration rate: 70 (51-81) *versus* 93 (83-106) mL/min/1.73m^2^, p=0.004. On day 1, D-dimer and fibrinogen levels were elevated but similar between groups: 1780 (1319-5517) *versus* 1794 (726-2324) ng/mL, p=0.145 and 608 (550-700) *versus* 642 (469-722) g/dL, p=0.95, respectively. Rotational thromboelastometry data were also similar between groups. However, antithrombin activity and protein C levels were lower in patients who developed acute kidney injury: 82 (75-92) *versus* 98 (90-116), p=0.028 and 70 (52-82) *versus* 88 (78-101) µ/mL, p=0.038, respectively. Mean protein C levels were lower in the group with acute kidney injury across multiple time points during their stay in the intensive care unit.

**Conclusion:**

Critically ill patients experiencing acute kidney injury exhibited lower endogenous anticoagulant levels. Further studies are needed to understand the role of natural anticoagulants in the pathophysiology of acute kidney injury within this population.

## In Brief

Silva et al. compared the coagulation profile in 30 critically ill COVID-19 patients with and without acute kidney injury. They demonstrated that both serum antithrombin activity and protein C levels, which are endogenous anticoagulants, were lower in patients who developed acute kidney injury

## Highlights

43.4% of the cohort developed acute kidney injury.

D-dimer and fibrinogen levels were high in both groups.

Rotational thromboelastometry data were similar between groups.

Serum levels of antithrombin activity and protein C were lower in patients who developed acute kidney injury.

## Graphical abstract

**Figure:**
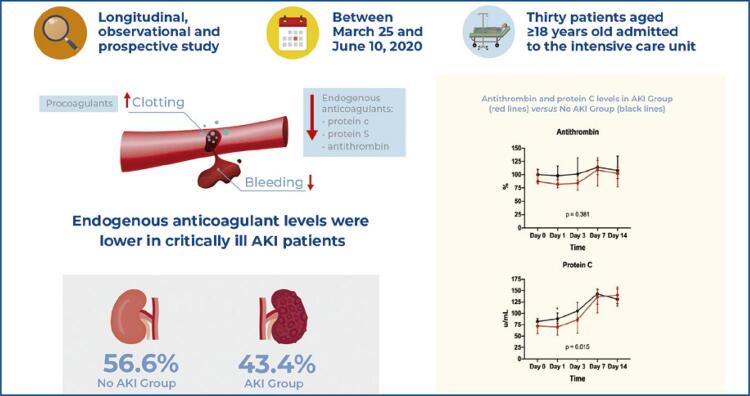

## INTRODUCTION

The severe acute respiratory syndrome coronavirus 2 (SARS-CoV-2) was first identified in Wuhan, China in early December 2019.^(1)^ This novel coronavirus has spread globally in a very short period of time, causing an international outbreak of severe acute respiratory syndrome known as corona virus disease (COVID-19). The first confirmed case of COVID-19 in Brazil was reported on February 26, 2020, in the city of São Paulo.^(2)^

Initial reports from China alleged that acute kidney injury (AKI) was not a frequent condition in patients with COVID-19.^(1,3-7)^ However, as the pandemic spread to Western countries, the incidence of AKI increased substantially,^(8-11)^ and the use of renal replacement therapy (RRT) reached up to 31% among critically ill patients,^(10)^ which is much higher than previously reported.^(12)^

Coagulopathy is a frequent condition in patients with COVID-19, and venous or arterial thrombotic manifestations have been extensively reported.^(13-15)^ To date, some data have shown that thrombus formation and vascular congestion might also affect glomerular and peritubular capillaries, which might be implicated in the pathophysiology of AKI in SARS-CoV-2 infection.^(16,17)^ Whether such findings are disease-specific or simply a sepsis epiphenomenon is still a matter of debate. Theoretically, an increased hypercoagulability state, fostering glomerular and tubular lesions, may explain the high incidence of AKI in critically ill patients with SARS-CoV-2 infection.

To date, no study has fully addressed coagulation abnormalities and the occurrence of AKI in severe patients with COVID-19 admitted to the intensive care unit (ICU). Conventional coagulation tests (CCT), platelet function, fibrinolysis, endogenous inhibitors of coagulation (antithrombin, protein C, and protein S) tests, and rotational thromboelastometry (ROTEM), which provides a real-time evaluation of clot formation kinetics, are altogether tests that could provide a more comprehensive understanding of the hypercoagulability state in COVID-19-associated AKI.

## OBJECTIVE

We aimed to evaluate the coagulation profiles of critically ill patients with COVID-19 who developed acute kidney injury, in comparison to critically ill patients who did not develop acute kidney injury during their stay in the intensive care unit.

## METHODS

### Study design

The present study is a secondary analysis of an original study designed to longitudinally address the coagulation, fibrinolysis, and endogenous anticoagulation systems of patients admitted to the ICU with severe COVID-19. The original study has been published elsewhere.^(18)^ This single-center, prospective observational longitudinal study, was conducted in the ICU of a private tertiary care hospital in São Paulo, Brazil, between March 25 and June 10, 2020.

The original study and this secondary analysis were approved by the Local Ethics Committee of *Hospital Israelita Albert Einstein* and by *Comissão Nacional de Ética em Pesquisa* (CONEP) with a waiver of informed consent (CAAE: 30175220.3.0000.0071; #3.937.833).

### Study population and group definition

Thirty patients aged ≥18 years old admitted to the ICU with a confirmed diagnosis of COVID-19 were included in this study. SARS-CoV-2 infection was confirmed by a positive reverse transcription-polymerase chain reaction (RT-PCR) assay.^(19)^

Exclusion criteria included chronic kidney disease (CKD) with a glomerular filtration rate (GFR) <30mL/min/1.73m^2^, pregnancy, known coagulopathy, current use of systemic anticoagulants or anti-platelet therapy or vitamin K antagonists, moribund patients, and patients who experienced cardiac arrest. Acute kidney injury was defined according to Kidney Disease: Improving Global Outcomes (KDIGO) criteria.^(20)^ Patients who developed any stage of AKI (KDIGO I, II, or III) were included in the “AKI Group” and the remaining patients (those without AKI) were included in the “No AKI Group”. Glomerular filtration rate (GFR) was estimated using the Chronic Kidney Disease Epidemiology Collaboration (CKD-EPI) formula.^(21)^

### Laboratory analysis

Laboratory tests were performed at baseline and on days 1, 3, 7, and 14 after study enrollment, except if the patient died or was discharged from the ICU.

### Conventional coagulation tests

Conventional coagulation tests included platelet count (XE 2100, Sysmex, São Paulo, Brazil), plasma fibrinogen concentration (Clauss method, Hemosil QFA thrombin, bovine), activated partial thromboplastin time (aPTT) (Hemosil Synthasil), prothrombin time (PT), and international normalized ratio (INR) (Hemosil PT-Fibrinogen HS Plus, IL Instrumentation Laboratory Company, Bedford MA, USA), and ionic calcium (ABL 800 FLEX, Radiometer Medical ApS, Copenhagen, Denmark).

### Platelet function test

Platelet function was assessed in whole blood samples using impedance aggregometry (The ROTEM^®^-Platelet- TEM Innovations GmbH, Munich Germany).^(22)^ Platelets were activated with arachidonic acid (ARATEM test) and adenosine diphosphate (ADPTEM).^(22)^

### Fibrinolysis and endogenous anticoagulation system

The following tests were used for assessing both fibrinolysis and endogenous anticoagulation system: D-dimer (Hemosil D-dimer HS 500 and Hemosil D-dimer HS 500 controls), serum plasminogen, alpha-2 antiplasmin (Plasmin Inhibitor), antithrombin, protein C, and free protein S (IL Instrumentation Laboratory Company, Bedford MA, USA).

### Rotational thromboelastometry

Rotational thromboelastometry analyses were performed using EXTEM (extrinsic coagulation pathway assessment), INTEM (intrinsic coagulation pathway assessment), and FIBTEM (extrinsic coagulation pathway assessment with additional platelet inhibition using Cytochalasin D) tests according to the manufacturer’s instructions.^(23)^ The following parameters were evaluated during ROTEM analysis: clotting time [CT- seconds (sec)], representing the time from the beginning of the test until clot firmness of 2mm; clot formation time (CFT- sec), representing the time between the detection of clot firmness of 2 and 20mm; and maximum clot firmness (MCF- mm), representing the highest amplitude of the thromboelastometric trace and indicating clot “strength”.^(24,25)^ROTEM tests were performed by laboratory technicians. Blood samples of approximately 3mL were collected by venipuncture into a tube with citrate (3.2%- Sarsted1, Wedel, Germany). Blood samples were processed within a maximum period of two hours for ROTEM analysis. The analyses were performed by pipetting 340μL of citrated whole blood and 20μL of 0.2M calcium chloride with specific activators into a cup. There was no change in methodology throughout the study period.^(25,26)^Hypercoagulability in ROTEM was defined as a reduction in clotting time (INTEM CT <100 sec or EXTEM CT <38 sec) or clot formation time (INTEM CFT <30 sec or EXTEM CFT <34 sec), and/or an increase in MCF (MCF INTEM or EXTEM MCF >72mm or FIBTEM MCF >25mm).^(26, 27)^

### Data collection

All study data were retrieved from the Epimed Monitor System (Epimed Solutions, Rio de Janeiro, Brazil), an electronic structured case report form where patient data are prospectively entered by trained ICU case managers.^(28)^Collected clinical variables included demographics, comorbidities, Simplified Acute Physiology score (SAPS 3 Score) at ICU admission,^(29)^Sequential Organ Failure Assessment score (SOFA Score)^(30)^ at ICU admission and on days 1, 3, 7, and 14 after study enrollment (unless the patient died or was discharged from the ICU), total maximum SOFA Score (from the time of study inclusion (baseline) up to 14 days after enrollment (unless the patient died or was discharged from the ICU),^(31)^ body mass index, treatment measures (*e.g*., macrolides, corticosteroids, interleukin-6 receptor antagonist, convalescent plasma, and lopinavir-ritonavir), supportive therapy (use of vasopressors, mechanical ventilation, noninvasive mechanical ventilation, and RRT during ICU stay), hospital length of stay (LOS) prior to ICU admission, ICU and hospital LOS, and ICU mortality. The time interval between ICU admission and AKI diagnosis (tAKI) was recorded in days.

The presence of thrombotic or hemorrhagic events and the use of prophylactic or therapeutic doses of low-molecular-weight heparin (LMWH) or unfractionated heparin (UFH) during ICU stay were recorded.

### Statistical analysis

Categorical variables were presented as n/n total (%). Continuous variables were presented as median and 25^th^-75^th^ percentile. Comparisons were performed between AKI and No AKI Groups. Categorical variables were compared between groups with Fisher’s exact test. Continuous variables were compared using independent Student’s *t*-test or Mann-Whitney U test in case of non-normal distribution, tested by the Kolmogorov-Smirnov test.

All laboratory tests performed on day 1 after enrollment were compared between groups. We have chosen this time point for comparisons between groups, as the mean time interval between ICU admission and AKI diagnosis (tAKI) was 1.7±1.1 days.

If a statistically significant difference was observed, a longitudinal assessment was performed using generalized estimating equations (GEE) with Groups (AKI and No AKI) and study time points (days 0, 1, 3, 7, and 14) as predictors. P values for group effect, time effect, and time-group interaction were presented.

Two-tailed tests were used. Statistical significance was set at p<0.05. No adjustment was made for missing data. SPSS™ version 26.0 was used for statistical analyses, and GraphPad Prism version 8.0.0 (GraphPad Software, San Diego, California, USA) was used for graph plotting.

## RESULTS

### Baseline characteristics

Thirty patients were included in this analysis. Out of those, 43.4% (13/30) were included in the AKI Group and 56.6% (17/30) in the No AKI Group (Table 1).

**Table 1:** Characteristics of study participants, administered treatments and clinical outcomes

Characteristics	AKI Group (n=13)	No AKI Group (n=17)	p value
Age, years (median, 25^th^-75^th^)	73 (60-84)	54 (47-64)	0.027^†^
Men, n (%)	7 (53.8)	8 (47.1)	0.71^‡^
BMI, kg/m^2^ (median, 25^th^-75^th^)	31 (25-35)	27 (24-31)	0.19^†^
Number of coexisting conditions, (median, 25^th^-75^th^)	3 (2-3)	1 (0-2)	<0.001^§^
Coexisting conditions, n (%)			
Hypertension	8 (61.5)	4 (23.5)	0.035^†^
*Diabetes mellitus*	9 (69.2)	2 (11.8)	0.002^£^
Obesity	7 (53.8)	5 (31.3)	0.21^‡^
Malignancy	1 (7.7)	3 (17.6)	0.61^£^
Heart failure	2 (15.4)	1 (5.9)	0.56^£^
COPD / Asthma	1 (7.7)	2 (11.8)	>0.99^£^
Chronic kidney disease	3 (23.1)	1 (5.9)	0.29^£^
Coronary artery disease	2 (15.4)	0 (0.0)	0.18^£^
SAPS III Score, points (median, 25^th^-75^th^)	48 (41-55)	49 (44-61)	0.69^†^
SOFA Score D0, points (median, 25^th^-75^th^)	7 (4-9)	5 (4-6)	0.19^†^
Maximum SOFA Score, points (median, 25^th^-75^th^)	13 (12-14)	7 (6-10)	<0.001^†^
Red blood cells transfusion (%)	30	0	0.01^‡^
Glomerular filtration rate, mL/min/1.73m^2^ (median, 25^th^-75^th^)			
Baseline	70 (51-81)	93 (85-106)	0.004^†^
At ICU admission	60 (35-77)	92 (80-104)	0.007^†^
COVID-19 therapy, n (%)			
Macrolides	11 (84.6)	17 (100.0)	0.18^£^
Glucocorticoids	11 (84.6)	14 (82.4)	0.86^£^
Convalescent plasma	4 (30.8)	6 (35.3)	>0.99^£^
Interleukin-6 receptor antagonist	0 (0.0)	3 (17.6)	0.23^£^
Lopinavir-ritonavir	1 (7.7)	1 (5.9)	>0.99^£^
Support during ICU stay, n (%)			
Renal replacement therapy	10 (76.9)	0	<0.001^£^
Vasopressors	13 (100.0)	14 (82.4)	0.23^£^
Mechanical ventilation	13 (100.0)	14 (82.4)	0.23^£^
Anticoagulants, n (%)			0.5^‡^
DVT prophylaxis	9 (69.2)	13 (76.5)	0.041^§^
Systemic anticoagulation	4 (30.8)	3 (17.6)	
Clinical outcomes			
Thrombosis, n (%)	4 (30.8)	2 (11.8)	0.36^£^
Bleeding, n (%)	0 (0.0)	3 (17.6)	0.23^£^
Died in ICU, n (%)	4 (30.8)	1 (5.9)	0.14^£^
ICU LOS, days (median, 25^th^-75^th^)	21 (16-42)	7 (6-16)	0.002^§^
Hospital LOS, days (median, 25^th^-75^th^)	32 (22-53)	17 (13-32)	0.066^§^

Values represent median (25^th^-75^th^ percentile) or n (%).

^†^ P values were calculated with the use of Independent Student’s *t*-test; ^‡^ χ^2^ test; ^§^ Mann-Whitney U test; ^£^ Fisher’s exact test.

AKI: acute kidney injury; BMI: body mass index; COPD: chronic obstructive pulmonary disease; SAPSIII: Simplified Acute Physiology Score III; SOFA: Sequential Organ Failure Assessment Score; ICU: intensive care unit; COVID-19: coronavirus disease 2019; LOS: length of stay; DVT: deep vein thrombosis; LOS: length of stay.

Patients in the AKI Group were older [73 (60-84) *versus* 54 (47-64) years, p=0.027, respectively for AKI and No AKI Groups], and hypertension (61.5 *versus* 23.5%, p=0.035, respectively) and *diabetes mellitus* (69.2 *versus* 11.8%, p=0.002, respectively) were more common among them.

Additionally, compared to no AKI patients, AKI patients had a lower baseline GFR at ICU admission [60 (35-77) *versus* 92 (80-104) mL/min/1.73m^2^, p=0.007] and presented a higher maximum SOFA Score during ICU stay [13 (12-14) *versus* 7 (6-10), p<0.001, respectively] (Table 1).

Overall, only four patients (13.3%) received red blood cell transfusions. No patient received platelet concentrate, FFP, cryoprecipitate, fibrinogen concentrate, PCC, or tranexamic acid during the study period.

### Administered treatment and organ support during ICU stay

Ten patients [10/13 (77%)] from the AKI Group received RRT, while no patient in the No AKI Group received RRT. The use of vasopressors, mechanical ventilation, specific treatments, and prophylactic or therapeutic anticoagulation did not differ between groups throughout the study duration (Table 1).

### Clinical outcomes

The incidence of bleeding, thrombosis, and ICU mortality did not differ between groups (Table 1). Intensive care unit length of stay was significantly higher in the AKI Group compared to the No AKI Group [21 (16-42) *versus* 7 (6-16) days, p=0.002] (Table 1).

### Laboratory and Conventional coagulation tests

While arterial pH was lower in the AKI Group compared to the No AKI Group [7.32 (7.25-7.39) *versus* 7.40 (7.39-7.43), respectively, p=0.010], ionized calcium and CCT (platelet count, PT, INR, aPTT, and plasma fibrinogen concentration) did not differ between groups (Table 2).

**Table 2:** Laboratorial, conventional coagulation, fibrinolysis and endogenous inhibitors of coagulation tests, performed at day 1 after enrollment

Parameters	Reference range	All Patients (n=30)	AKI Group (n=13)	No AKI Group (n=17)	p value
Laboratory tests					
Arterial pH	7.35-7.45	7.38 (7.31-7.40)	7.32 (7.25-7.39)	7.40 (7.39-7.43)	0.010^†^
Ionized calcium (mmol/L)	1.14-1.31	1.15 (1.11-1.20)	1.18 (1.10-1.22)	1.15 (1.12-1.19)	0.9^†^
Hemoglobin (g/dL)	13.5-17.5	11.4 (10.2-12.2)	11.2 (10.1-13.3)	11.5 (10.7-12.1)	0.99^†^
Conventional coagulation tests					
Platelets (x10^9^/L)	150-450	236 (182-268)	264 (178-268)	225 (208-258)	0.74^§^
Prothrombin time (sec)	70-100	81 (69-89)	87 (69-99)	79 (75-85)	0.96^†^
INR	0.96-1.30	1.14 (1.07-1.25)	1.08 (1.01-1.26)	1.15 (1.10-1.19)	0.71^§^
aPTT (sec)	25.6-35.5	28.3 (25.6-32.2)	28.3 (25.6-32.8)	27.3 (25.6-32.8)	0.59^†^
Fibrinogen (g/dL)	200-400	642 (470-722)	608 (550-700)	642 (469-722)	0.95^†^
Fibrinolysis					
D-dimer (ng/mL)	<500	1787 (762-4048)	1780 (1319-5517)	1794 (726-2324)	0.145^§^
Plasminogen (%)	80-132	86 (74-96)	78 (70-89)	90 (77-107)	0.22^†^
Alpha-2 antiplasmin (%)	98-122	127 (112-139)	121 (105-129)	132 (122-143)	0.071^†^
Endogenous anticoagulation					
Antithrombin (%)	75-110	92 (79-109)	82 (75-92)	98 (90-116)	0.028^†^
Protein C (u/mL)	60-130	81 (66-92)	70 (52-82)	88 (78-101)	0.038^†^
Protein S (u/mL)	55-140	28 (20-48)	32 (18-35)	26 (21-51)	0.45^§^

Values represent median (25^th^-75^th^ percentile).

^†^ P values were calculated with the use of independent Student’s *t*-test; ^§^ Mann-Whitney U test.

INR: international normalized ratio; aPTT: activated partial thromboplastin time; AKI: acute kidney injury.

According to rotational thromboelastometry, hypercoagulability was observed in 29 patients, and no differences were observed between the AKI and No AKI Groups 13/13 (100%) *versus* 16/17 (94%) patients, p=1.000 (Table 3). Additionally, rotational thromboelastometry and platelet function tests were similar between the AKI and No AKI Groups. Maximum clot firmness in FIBTEM was high in both groups (Table 3).

**Table 3:** Rotational thromboelastometry and platelet function test, performed on day 1 after enrollment

Parameters	Reference range	All Patients (n=30)	AKI Group (n=13)	No AKI Group (n=17)	p value
ROTEM-INTEM					
Clotting time (sec)	100-240	160 (156-191)	160 (151-185)	159 (156-191)	0.93^§^
Clot formation time (sec)	30-110	47 (42-57)	46 (43-55)	52 (41-57)	0.93^§^
Maximum clot firmness (mm)	50-72	71 (69-73)	72 (70-73)	70 (69-73)	0.27^†^
Maximum lysis (%)	<5	8 (5-10)	7 (6-10)	8 (4-10)	0.71^†^
ROTEM-EXTEM					
Clotting time (sec)	38-79	73 (66-88)	72 (67-77)	73 (66-88)	0.64^†^
Clot formation time (sec)	34-159	56 (45-64)	55 (45-60)	56 (45-64)	0.9^§^
Maximum clot firmness (mm)	50-72	73 (70-75)	73 (70-75)	73 (68-74)	0.5^†^
Maximum lysis (%)	<15	9 (6-13)	10 (7-13)	9 (6-11)	0.46^†^
ROTEM-FIBTEM					
Maximum clot firmness (mm)	9-25	37 (30-40)	38 (31-40)	36 (29-40)	0.35^†^
Patients with hypercoagulability state in ROTEM analysis		29/30	13/13	16/17	>0.99^£^
PLATELET function test					
ARATEM test (sec)	70-153	87 (58-113)	87 (58-113)	87 (60-110)	0.64^†^
ADPTEM test (sec)	56-139	105 (72-128)	111 (71-141)	102 (78-118)	0.47^†^

Values represent median (25^th^-75^th^ percentile).

^†^ P values were calculated with the use of (a) Independent Student’s *t*-test, ^§^ Mann-Whitney U test; ^£^ Fisher’s exact test.

AKI: acute kidney injury.

Maximum lysis levels in both INTEM and EXTEM were within the reference range in both AKI and No AKI Groups (Table 3). D-dimer levels were 3- to 4-fold higher than the upper reference range limit in both groups (Table 2). Plasminogen and alpha-2 antiplasmin levels did not differ between groups (Table 2).

**Figure 1:**
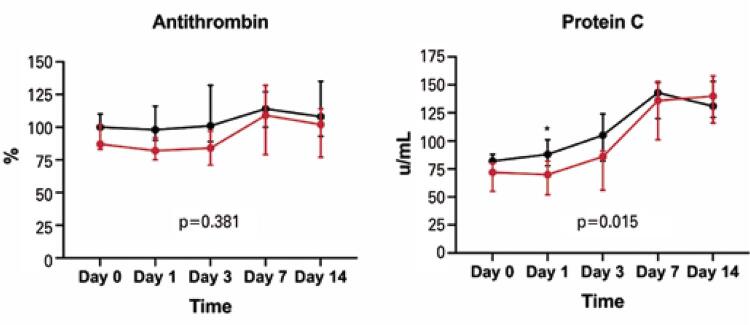
Red lines denote Acute Kidney Injury Group and black lines No Acute Kidney Injury Group * Comparison significant at the 0.05 level Acute Kidney Injury Group *versus* No Acute Kidney Injury Group. Values represent median interquartile range. P values represent time-group interaction calculated with the use of generalized estimating equations.

Endogenous anticoagulants, represented by antithrombin activity [82 (75-92) *versus* 98 (90-116), p=0.028, respectively], and protein C levels [70 (52-82) *versus* 88 (78-101) µ/mL, p=0.038, respectively] were lower in patients who developed AKI compared to those in the No AKI Group (Table 2 and Figure 1). Protein S concentration was similar between groups.

## DISCUSSION

In this study, we found that a hypercoagulability state was frequently observed in critically ill patients with COVID-19, regardless of AKI development. Furthermore, we observed a lower antithrombin activity and reduced plasma protein C levels (endogenous inhibitors of coagulation) in critically ill COVID-19 patients who developed AKI during their ICU stay compared to patients without an AKI diagnosis.

Acute kidney injury in critically ill patients with COVID-19 exhibits distinct clinical features compared to those previously reported in epidemiological studies.^(32,33)^ Acute kidney injury is more prevalent in this population,^(8-10)^ and its onset is closely associated with the worsening of respiratory failure.^(8)^ Moreover, severe circulatory shock is not common in these patients.^(34)^ Kidney injury caused by COVID-19 is mediated locally by direct viral interaction with angiotensin-converting enzyme receptors in kidney tissue and systemically by inflammatory cell recruitment, released from lung injury. Pulmonary damage-associated and pathogen-associated molecular patterns lead to increased cytokine release, which is associated with systemic inflammation and kidney injury.^(35-37)^ Histopathological studies of kidney tissues have revealed specific findings, such as diffuse acute tubular injury with cytoplasmic vacuoles in proximal tubules,^(16)^ erythrocyte aggregation, and fibrin thrombi, resulting in vascular congestion/obstruction of glomerular tufts and peritubular capillaries, as well as glomerular ischemia.^(16,17)^ These latter findings suggest that SARS-CoV-2 infection may predispose individuals to thrombotic microangiopathy in the kidneys. Collectively, these factors may indicate a distinct AKI pathophysiology.^(38)^

SARS-CoV-2 infection has been linked to a high incidence of thrombotic events. Recently, Klok et al. demonstrated that symptomatic acute pulmonary embolism, deep vein thrombosis, ischemic stroke, myocardial infarction, or systemic arterial embolism were observed in 31% of patients in the ICU.^(13)^ It remains unknown whether similar manifestations could affect the kidneys, leading to disease-specific AKI.

The AKI incidence in the present study was relatively high (41% of the study population), aligning with other studies from Brazil,^(39,40)^ and the United States.^(8,41,42)^ Additionally, 20% of the overall cohort experienced thrombotic events, and hypercoagulability in ROTEM analysis was found in nearly all patients (97% of the overall cohort). Furthermore, D-dimer and fibrinogen levels were extremely high. However, the most interesting finding was the lower levels of antithrombin activity and protein C at AKI diagnosis. Moreover, protein C levels exhibited a significant time-group interaction, as they were lower across multiple time points in the AKI Group compared to the No AKI Group.

Antithrombin is an endogenous inhibitor of coagulation that neutralizes several coagulation enzymes, such as thrombin, plasmin, and coagulation factors IXa, Xa, XIa, XIIa.^(43)^ Thrombin cleaves antithrombin, which subsequently traps the thrombin molecule, generating an enzyme-inhibitor complex that is eventually removed from the bloodstream.^(44)^ In addition to its antithrombotic effect, antithrombin possesses anti-inflammatory properties, as it blocks thrombin-induced inflammatory pathways and inhibits Xa-induced production of interleukins 6, 8, E-selectin, and other molecules involved in monocyte recruitment and adhesion to endothelial cells.^(44)^

Lower levels of antithrombin activity in patients who developed AKI may suggest higher antithrombin consumption to remove thrombin from circulation. There are several possible explanations for these findings in patients with AKI: first, lower antithrombin activity levels could reflect more severe systemic disease. However, the SAPSIII Score and the lowest PO_2_/FiO_2_ ratio were similar between groups, indicating comparable organic and pulmonary dysfunction. A second possibility is that lower antithrombin activity could simply be a marker of poor clinical conditions, as lower antithrombin levels are associated with older age,^(45,46)^particularly in male patients,^(45)^ and with *diabetes mellitus*.^(47)^ Patients who developed AKI were older and more frequently diagnosed with *diabetes mellitus*, pathophysiological states characterized by substantial endothelial changes. A third possibility is that viral infection in some patients increases thrombin generation, leading to higher antithrombin consumption. In this case, kidney injury could be a consequence of higher thrombin levels (potentially causing the previously mentiones thrombotic lesions in glomerular and peritubular capillaries). In summary, lower antithrombin levels have been associated with AKI, particularly in systemic inflammatory conditions such as sepsis-associated AKI,^(48,49)^ preeclampsia^(50)^ and solid organ transplantation.^(51)^

The potential link between lower antithrombin activity and AKI remains unclear, but growing evidence from clinical and experimental data suggests such an association, as lower antithrombin activity levels have been recently linked with AKI in elderly septic patients and after cardiac surgery.^(49,52)^ Additionally, heterozygous knockout rats for the *SerpinC1* gene (which encodes antithrombin) developed AKI following a renal ischemia/reperfusion injury model.^(52)^ Moreover, in sepsis, reduced activity of endogenous coagulation inhibitors such as antithrombin, protein C, and S leads to a procoagulant condition.^(53)^ Decreased levels of these inhibitors could contribute to prothrombotic and inflammatory conditions, potentially setting the stage for AKI development. Indeed, lower protein C levels have been associated with AKI in critically ill patients.^(54)^

Interestingly, ROTEM analysis did reflect lower endogenous anticoagulant levels, as hypercoagulability state was detected in 29 patients. Maximum clot firmness in the FIBTEM test was high in virtually all patients but failed to predict AKI in this population.

It is essential to note that other factors might play a role in COVID-19-associated AKI: severe pulmonary injury itself could lead to hypoxemia and inflammation, which are associated with AKI.^(38)^ Additionally, increased thoracic pressure and venous congestion induced by mechanical ventilation may also contribute to AKI.^(55)^

This study has some limitations: it was a single-center study with a relatively small sample size. Furthermore, an observational study cannot establish a causal link between AKI and lower levels of endogenous coagulation inhibitors. However, a fully comprehensive coagulation profile analysis, performed at multiple time points in a specific population, is undoubtedly a strength of this study. The consistent finding of lower protein C levels in the AKI Group across time points (and not just a single one) reduces the possibility of bias in this observation.

## CONCLUSION

In conclusion, endogenous anticoagulants, particularly antithrombin activity and protein C levels, were lower in critically ill COVID-19 patients who developed acute kidney injury. The pathophysiological causes of acute kidney injury in COVID-19 patients are multifaceted and complex, and coagulation disorders, such as a diminished antifibrinolytic profile, may increase the risk of acute kidney injury development in this population. Further studies are needed to fully establish a causal link for this finding.

## References

[1] Huang C, Wang Y, Li X, Ren L, Zhao J, Hu Y (2020). Clinical features of patients infected with 2019 novel coronavirus in Wuhan, China. Lancet.

[2] Serdan TD, Masi LN, Gorjao R, Pithon-Curi TC, Curi R, Hirabara SM (2020). COVID-19 in Brazil: historical cases, disease milestones, and estimated outbreak peak. Travel Med Infect Dis.

[3] Wang L, Li X, Chen H, Yan S, Li D, Li Y (2020). Coronavirus Disease 19 Infection Does Not Result in Acute Kidney Injury: an Analysis of 116 Hospitalized Patients from Wuhan, China. Am J Nephrol.

[4] Chen N, Zhou M, Dong X, Qu J, Gong F, Han Y (2020). Epidemiological and clinical characteristics of 99 cases of 2019 novel coronavirus pneumonia in Wuhan, China: a descriptive study. Lancet.

[5] Guan WJ, Ni ZY, Hu Y, Liang WH, Ou CQ, He JX, Liu L, Shan H, Lei CL, Hui DSC, Du B, Li LJ, Zeng G, Yuen KY, Chen RC, Tang CL, Wang T, Chen PY, Xiang J, Li SY, Wang JL, Liang ZJ, Peng YX, Wei L, Liu Y, Hu YH, Peng P, Wang JM, Liu JY, Chen Z, Li G, Zheng ZJ, Qiu SQ, Luo J, Ye CJ, Zhu SY, Zhong NS, China Medical Treatment Expert Group for Covid-19 (2020). Clinical Characteristics of Coronavirus Disease 2019 in China. N Engl J Med.

[6] Shi S, Qin M, Shen B, Cai Y, Liu T, Yang F (2020). Association of Cardiac Injury With Mortality in Hospitalized Patients With COVID-19 in Wuhan, China. JAMA Cardiol.

[7] Hu L, Chen S, Fu Y, Gao Z, Long H, Ren HW (2020). Risk Factors Associated With Clinical Outcomes in 323 Coronavirus Disease 2019 (COVID-19) Hospitalized Patients in Wuhan, China. Clin Infect Dis.

[8] Richardson S, Hirsch JS, Narasimhan M, Crawford JM, McGinn T, Davidson KW, the Northwell COVID-19 Research Consortium (2020). Presenting Characteristics, Comorbidities, and Outcomes Among 5700 Patients Hospitalized With COVID-19 in the New York City Area. JAMA.

[9] Cummings MJ, Baldwin MR, Abrams D, Jacobson SD, Meyer BJ, Balough EM (2020). Epidemiology, clinical course, and outcomes of critically ill adults with COVID-19 in New York City: a prospective cohort study. Lancet.

[10] Doher MP, Torres de Carvalho FR, Scherer PF, Matsui TN, Ammirati AL, Silva BC (2021). Acute Kidney Injury and Renal Replacement Therapy in Critically Ill COVID-19 Patients: Risk Factors and Outcomes: a Single-Center Experience in Brazil. Blood Purif.

[11] Whittaker SA, Fuchs BD, Gaieski DF, Christie JD, Goyal M, Meyer NJ (2015). Epidemiology and outcomes in patients with severe sepsis admitted to the hospital wards. J Crit Care.

[12] Helms J, Tacquard C, Severac F, Leonard-Lorant I, Ohana M, Delabranche X, Merdji H, Clere-Jehl R, Schenck M, Fagot Gandet F, Fafi-Kremer S, Castelain V, Schneider F, Grunebaum L, Anglés-Cano E, Sattler L, Mertes PM, Meziani F, CRICS TRIGGERSEP Group (Clinical Research in Intensive Care and Sepsis Trial Group for Global Evaluation and Research in Sepsis) (2020). High risk of thrombosis in patients with severe SARS-CoV-2 infection: a multicenter prospective cohort study. Intensive Care Med.

[13] Klok FA, Kruip MJ, van der Meer NJ, Arbous MS, Gommers DA, Kant KM (2020). Incidence of thrombotic complications in critically ill ICU patients with COVID-19. Thromb Res.

[14] Panigada M, Bottino N, Tagliabue P, Grasselli G, Novembrino C, Chantarangkul V (2020). Hypercoagulability of COVID-19 patients in intensive care unit: a report of thromboelastography findings and other parameters of hemostasis. J Thromb Haemost.

[15] Su H, Yang M, Wan C, Yi LX, Tang F, Zhu HY (2020). Renal histopathological analysis of 26 postmortem findings of patients with COVID-19 in China. Kidney Int.

[16] Duarte-Neto AN, Monteiro RA, Silva LF, Malheiros DM, Oliveira EP, Theodoro-Filho J (2020). Pulmonary and systemic involvement in COVID-19 patients assessed with ultrasound-guided minimally invasive autopsy. Histopathology.

[17] Corrêa TD, Cordioli RL, Guerra JC, Silva BC, Rodrigues RR, Souza GM (2020). Coagulation profile of COVID-19 patients admitted to the ICU: an exploratory study. PLoS One.

[18] Corman VM, Landt O, Kaiser M, Molenkamp R, Meijer A, Chu DK (2020). Detection of 2019 novel coronavirus (2019-nCoV) by real-time RT-PCR. Euro Surveill.

[19] (2012). Section 2: Definition AK. Section 2: AKI Definition. Kidney Int Suppl.

[20] Levey AS, Stevens LA, Schmid CH, Zhang YL, Castro AF, Feldman HI, Kusek JW, Eggers P, Van Lente F, Greene T, Coresh J, CKD-EPI (Chronic Kidney Disease Epidemiology Collaboration) (2009). A new equation to estimate glomerular filtration rate. Ann Intern Med.

[21] Petricevic M, Konosic S, Biocina B, Dirkmann D, White A, Mihaljevic MZ (2016). Bleeding risk assessment in patients undergoing elective cardiac surgery using ROTEM(^®^) platelet and Multiplate(^®^) impedance aggregometry. Anaesthesia.

[22] Whiting D, DiNardo JA (2014). TEG and ROTEM: technology and clinical applications. Am J Hematol.

[23] Lier H, Vorweg M, Hanke A, Görlinger K (2013). Thromboelastometry guided therapy of severe bleeding. Essener Runde algorithm. Hamostaseologie.

[24] Crochemore T, Piza FM, Rodrigues RD, Guerra JC, Ferraz LJ, Corrêa TD (2017). A new era of thromboelastometry. einstein (Sao Paulo).

[25] Crochemore T, Corrêa TD, Lance MD, Solomon C, Neto AS, Guerra JC (2018). Thromboelastometry profile in critically ill patients: a single-center, retrospective, observational study. PLoS One.

[26] Lang T, Bauters A, Braun SL, Pötzsch B, von Pape KW, Kolde HJ (2005). Multi-centre investigation on reference ranges for ROTEM thromboelastometry. Blood Coagul Fibrinolysis.

[27] Zampieri FG, Soares M, Borges LP, Salluh JI, Ranzani OT (2017). The Epimed Monitor ICU Database^®^: a cloud-based national registry for adult intensive care unit patients in Brazil. Rev Bras Ter Intensiva.

[28] Moreno RP, Metnitz PG, Almeida E, Jordan B, Bauer P, Campos RA, Iapichino G, Edbrooke D, Capuzzo M, Le Gall JR, SAPS 3 Investigators (2005). SAPS 3--From evaluation of the patient to evaluation of the intensive care unit. Part 2: Development of a prognostic model for hospital mortality at ICU admission. Intensive Care Med.

[29] Vincent JL, Moreno R, Takala J, Willatts S, De Mendonça A, Bruining H (1996). The SOFA (Sepsis-related Organ Failure Assessment) score to describe organ dysfunction/failure. On behalf of the Working Group on Sepsis-Related Problems of the European Society of Intensive Care Medicine. Intensive Care Med.

[30] Moreno R, Vincent JL, Matos R, Mendonça A, Cantraine F, Thijs L (1999). The use of maximum SOFA score to quantify organ dysfunction/failure in intensive care. Results of a prospective, multicentre study. Working Group on Sepsis related Problems of the ESICM. Intensive Care Med.

[31] Hoste EA, Bagshaw SM, Bellomo R, Cely CM, Colman R, Cruz DN (2015). Epidemiology of acute kidney injury in critically ill patients: the multinational AKI-EPI study. Intensive Care Med.

[32] Uchino S, Kellum JA, Bellomo R, Doig GS, Morimatsu H, Morgera S, Schetz M, Tan I, Bouman C, Macedo E, Gibney N, Tolwani A, Ronco C, Beginning and Ending Supportive Therapy for the Kidney (BEST Kidney) Investigators (2005). Acute renal failure in critically ill patients: a multinational, multicenter study. JAMA.

[33] Hirsch JS, Ng JH, Ross DW, Sharma P, Shah HH, Barnett RL, Hazzan AD, Fishbane S, Jhaveri KD, Northwell COVID-19 Research Consortium, Northwell Nephrology COVID-19 Research Consortium (2020). Acute kidney injury in patients hospitalized with COVID-19. Kidney Int.

[34] Richardson S, Hirsch JS, Narasimhan M, Crawford JM, McGinn T, Davidson KW, Barnaby DP, Becker LB, Chelico JD, Cohen SL, Cookingham J, Coppa K, Diefenbach MA, Dominello AJ, Duer-Hefele J, Falzon L, Gitlin J, Hajizadeh N, Harvin TG, Hirschwerk DA, Kim EJ, Kozel ZM, Marrast LM, Mogavero JN, Osorio GA, Qiu M, Zanos TP, the Northwell COVID-19 Research Consortium (2020). Presenting Characteristics, Comorbidities, and Outcomes Among 5700 Patients Hospitalized With COVID-19 in the New York City Area. JAMA.

[35] Zaim S, Chong JH, Sankaranarayanan V, Harky A (2020). COVID-19 and Multiorgan Response. Curr Probl Cardiol.

[36] Sharma P, Uppal NN, Wanchoo R, Shah HH, Yang Y, Parikh R, Khanin Y, Madireddy V, Larsen CP, Jhaveri KD, Bijol V, Northwell Nephrology COVID-19 Research Consortium (2020). COVID-19-Associated Kidney Injury: a Case Series of Kidney Biopsy Findings. J Am Soc Nephrol.

[37] Legrand M, Bell S, Forni L, Joannidis M, Koyner JL, Liu K (2021). Pathophysiology of COVID-19-associated acute kidney injury. Nat Rev Nephrol.

[38] Głowacka M, Lipka S, Młynarska E, Franczyk B, Rysz J (2021). Acute Kidney Injury in COVID-19. Int J Mol Sci.

[39] Batlle D, Soler MJ, Sparks MA, Hiremath S, South AM, Welling PA, Swaminathan S, COVID-19 and ACE2 in Cardiovascular, Lung, and Kidney Working Group (2020). Acute Kidney Injury in COVID-19: Emerging Evidence of a Distinct Pathophysiology. J Am Soc Nephrol.

[40] Costa RL, Sória TC, Salles EF, Gerecht AV, Corvisier MF, Menezes MA (2021). Acute kidney injury in patients with Covid-19 in a Brazilian ICU: incidence, predictors and in-hospital mortality. J Bras Nefrol.

[41] Chan L, Chaudhary K, Saha A, Chauhan K, Vaid A, Zhao S, Paranjpe I, Somani S, Richter F, Miotto R, Lala A, Kia A, Timsina P, Li L, Freeman R, Chen R, Narula J, Just AC, Horowitz C, Fayad Z, Cordon-Cardo C, Schadt E, Levin MA, Reich DL, Fuster V, Murphy B, He JC, Charney AW, Böttinger EP, Glicksberg BS, Coca SG, Nadkarni GN, on behalf of the Mount Sinai COVID Informatics Center (MSCIC) (2021). AKI in Hospitalized Patients with COVID-19. J Am Soc Nephrol.

[42] Fisher M, Neugarten J, Bellin E, Yunes M, Stahl L, Johns TS (2020). AKI in Hospitalized Patients with and without COVID-19: A Comparison Study. J Am Soc Nephrol.

[43] Blajchman MA (1994). An overview of the mechanism of action of antithrombin and its inherited deficiency states. Blood Coagul Fibrinolysis.

[44] Levy JH, Sniecinski RM, Welsby IJ, Levi M (2016). Antithrombin: anti-inflammatory properties and clinical applications. Thromb Haemost.

[45] Zhu T, Ding Q, Bai X, Wang X, Kaguelidou F, Alberti C (2011). Normal ranges and genetic variants of antithrombin, protein C and protein S in the general Chinese population. Results of the Chinese Hemostasis Investigation on Natural Anticoagulants Study I Group. Haematologica.

[46] Franchi F, Biguzzi E, Martinelli I, Bucciarelli P, Palmucci C, D’Agostino S (2013). Normal reference ranges of antithrombin, protein C and protein S: effect of sex, age and hormonal status. Thromb Res.

[47] Hernández-Espinosa D, Ordóñez A, Miñano A, Martínez-Martínez I, Vicente V, Corral J (2009). Hyperglycaemia impairs antithrombin secretion: possible contribution to the thrombotic risk of diabetes. Thromb Res.

[48] Xie Y, Zhang Y, Tian R, Jin W, Du J, Zhou Z (2021). A prediction model of sepsis-associated acute kidney injury based on antithrombin III. Clin Exp Med.

[49] Xie Y, Tian R, Jin W, Xie H, Du J, Zhou Z (2020). Antithrombin III expression predicts acute kidney injury in elderly patients with sepsis. Exp Ther Med.

[50] Samejima T, Yamashita T, Takeda Y, Adachi T (2021). Low antithrombin levels accompanied by high urine protein/creatinine ratios are predictive of acute kidney injury among CS patients with preeclampsia. J Matern Fetal Neonatal Med.

[51] Park J, Cho S, Cho YJ, Choi HJ, Hong SH, Chae MS (2021). Predictive Utility of Antithrombin III in Acute Kidney Injury in Living-Donor Liver Transplantation: a Retrospective Observational Cohort Study. Transplant Proc.

[52] Wang F, Zhang G, Lu Z, Geurts AM, Usa K, Jacob HJ (2015). Antithrombin III/SerpinC1 insufficiency exacerbates renal ischemia/reperfusion injury. Kidney Int.

[53] Martínez MA, Peña JM, Fernández A, Jiménez M, Juárez S, Madero R (1999). Time course and prognostic significance of hemostatic changes in sepsis: relation to tumor necrosis factor-alpha. Crit Care Med.

[54] Bouchard J, Malhotra R, Shah S, Kao YT, Vaida F, Gupta A (2015). Levels of protein C and soluble thrombomodulin in critically ill patients with acute kidney injury: a multicenter prospective observational study. PLoS One.

[55] Joannidis M, Forni LG, Klein SJ, Honore PM, Kashani K, Ostermann M (2020). Lung-kidney interactions in critically ill patients: consensus report of the Acute Disease Quality Initiative (ADQI) 21 Workgroup. Intensive Care Med.

